# Rhizosphere Soil Microbial Community Under Ice in a High-Latitude Wetland: Different Community Assembly Processes Shape Patterns of Rare and Abundant Microbes

**DOI:** 10.3389/fmicb.2022.783371

**Published:** 2022-05-23

**Authors:** Jiaming Ma, Kang Ma, Jingling Liu, Nannan Chen

**Affiliations:** State Key Laboratory of Water Environment Simulation, School of Environment, Beijing Normal University, Beijing, China

**Keywords:** bacterial and fungal communities, seasonally ice-covered, community assembly, temporal dynamic, rare taxa, iCAMP package

## Abstract

The rhizosphere soil microbial community under ice exhibits higher diversity and community turnover in the ice-covered stage. The mechanisms by which community assembly processes shape those patterns are poorly understood in high-latitude wetlands. Based on the 16S rRNA gene and ITS sequencing data, we determined the diversity patterns for the rhizosphere microbial community of two plant species in a seasonally ice-covered wetland, during the ice-covered and ice-free stages. The ecological processes of the community assembly were inferred using the null model at the phylogenetic bins (taxonomic groups divided according to phylogenetic relationships) level. Different effects of ecological processes on rare and abundant microbial sub-communities (defined by the relative abundance of bins) and bins were further analyzed. We found that bacterial and fungal communities had higher alpha and gamma diversity under the ice. During the ice-free stage, the dissimilarity of fungal communities decreased sharply, and the spatial variation disappeared. For the bacterial community, homogeneous selection, dispersal limitation, and ecological processes (undominated processes) were the main processes, and they remained relatively stable across all stages. For the fungal community, during the ice-covered stage, dispersal limitation was the dominant process. In contrast, during the ice-free stage, ecological drift processes were more important in the *Scirpus* rhizosphere, and ecological drift and homogeneous selection processes were more important in the *Phragmites* rhizosphere. Regarding the different effects of community assembly processes on abundant and rare microbes, abundant microbes were controlled more by homogeneous selection. In contrast, rare microbes were controlled more by ecological drift, dispersal limitation, and heterogeneous selection, especially bacteria. This is potentially caused by the low growth rates or the intermediate niche breadths of rare microbes under the ice. Our findings suggest the high diversity of microbial communities under the ice, which deepens our understanding of various ecological processes of community assembly across stages and reveals the distinct effects of community assembly processes on abundant and rare microbes at the bin level.

## Introduction

It is increasingly recognized that microorganisms can exist and grow under the ice (Tran et al., 2018). For microbial communities in seasonally ice-covered habitats, the ice-covered stage is still a poorly studied period compared to the ice-free stage (Jansen et al., 2021). Some studies have found that bacterial diversity increases at sub-zero temperatures in controlled experiments (Juan et al., 2018), while other studies found that soil bacterial diversity decreased (Zhang et al., 2017) or increased at first and then decreased during freeze-thaw in the forest (Sang et al., 2021). In wetlands located in high-latitude regions where the soil is seasonally covered by ice or water rather than snow or air, the plant inputs can change dramatically, potentially differentiating wetland bacterial diversity from other habitats. The environment under the ice may have two opposing effects on the alpha diversity of bacteria. Although the more selective environment under the ice (low temperature, low light) may decrease the alpha diversity (Butler et al., 2019), the bacteria are believed to be mainly dormant under the ice, and dormancy could increase alpha diversity, thereby preventing bacterial species from going extinct (Butler et al., 2019). Thus, it remains uncertain whether bacterial communities have higher alpha diversity under the ice in wetlands. Fungi are another important component of the microbial community. Though some studies have shown that the fungal: bacterial biomass ratios may increase in the low-temperature environment (Robroek et al., 2013), studies on fungal diversity under the ice are even more sparse. Therefore, more evidence on the diversity patterns of microbial communities, especially the fungal community, across ice-covered seasons in wetlands needs to be investigated.

The diversity and biogeography patterns of microbial communities are shaped by many processes. Niche-based theory suggests that non-random and niche-based ecological processes govern diversity patterns, and these are known as deterministic processes (Chesson, 2000). On the other hand, neutral theory suggests that ecological processes, where all species have equivalent fitness, govern the diversity, and these processes are termed stochastic processes (Chave, 2004). It was gradually recognized that the deterministic and stochastic processes jointly govern the diversity patterns (Chase and Myers, 2011). To integrate these two processes that are based on different theories into a coherent framework, the community assembly processes can be divided into four fundamental ecological processes: selection (including environmental filtering, biotic interactions, and host filtering), dispersal, ecological drift (random birth and death), and diversification (speciation and extinction) (Vellend, 2016; Zhou and Ning, 2017; Ning et al., 2020).

The influence of environmental filtering (one of the selection factors) on bacterial communities under the ice has been studied, specifically relating to dissolved oxygen (Bertilsson et al., 2013), low light, and temperature (Cruaud et al., 2020). Host filtering of plants is also an important factor in selection. Microbiomes in the rhizosphere are considered highly affected by rhizo-deposits and are in higher abundance and activity than in bulk soil (Prashar et al., 2014). The species of plants play an important role in affecting rhizosphere microbial community assembly processes (Philippot et al., 2013; Fitzpatrick et al., 2018; Zhalnina et al., 2018; Matthews et al., 2019). The different development stages of plants could also influence the composition and the community assembly processes of the rhizosphere microbial community (Chaparro et al., 2014; Bell et al., 2015).

Most studies related to the rhizosphere microbial community have concentrated on the various stages of plant growth (Bell et al., 2015), including seedling, growth, and flowering (Chaparro et al., 2014), but similar studies during the non-growing season (e.g., the ice-covered stage) are limited. Some species of plants will stop growing and partially or entirely wither during the ice-covered season. A decrease in photosynthesis may dampen the secretory activity of the roots, thereby affecting the community assembly processes of the rhizosphere microbial community (He et al., 2020). Additionally, other ecological processes, such as dispersal and ecological drift of microbial communities, under the ice have also been less well-studied. To further understand how different ecological processes work together, it is necessary to quantify their relative importance. The null model analysis is a useful tool that uses randomization procedures to quantify these ecological processes and may provide an understanding of the relationship between changes in the environment and the microbial community (Hanson et al., 2012; Stegen et al., 2012, 2013; Zhou and Ning, 2017). This approach can be used to study and quantify the community assembly processes of the soil microbial communities that live in different plant rhizospheres under the ice.

The relative abundance of microbial taxa may play an important role in regulating different community assembly processes, for example, low abundance may enhance the relative importance of ecological drift (Nemergut et al., 2013). In natural ecosystems, the vast majority of microbial taxa have a low relative abundance of rare microbial taxa, which are driven by different processes than the abundant microbial taxa (Magurran and Henderson, 2003; Martinez et al., 2015; Jousset et al., 2017). The drivers of microbial community rarity include dispersal (Lee et al., 2021), narrow niche breadths (Jiao and Lu, 2020), low growth rates (Liao et al., 2017; Lee et al., 2021), and biotic interactions (Jousset et al., 2017). During the ice-covered season, those drivers change significantly, including the dispersal medium, growth ratio, and nutrient inputs, leading to changes in species rarity. Recent studies researched the relationship between community assembly processes and the relative abundance of microbial taxa by dividing the microbial community into abundant, intermediate, and rare microbial sub-communities according to an artificially selected threshold of relative abundance (Liao et al., 2017; Jiao and Lu, 2020; Lee et al., 2021; Wan et al., 2021; Zheng et al., 2021). However, it is important to note that the levels of division (sub-community) and the threshold of division (artificially selected) may influence the study results. A newly published phylogenetic bin-based null model (the “Icamp” model) provides an approach to solve this issue (Ning et al., 2020). It calculates ecological processes of community assembly at the taxonomic group level (hereafter, the term “bins” is used to represent these taxa groups) (Ning et al., 2020). Many recent studies use this framework (Ceja-Navarro et al., 2021; Dong et al., 2021; Le Roux et al., 2021; Sun C. et al., 2021; Sun Y. et al., 2021). The influence of ecological processes on microbes with different levels of abundance (abundant, intermediate, and rare) could be further analyzed at the level of bins using this framework to explore the underlying mechanisms.

This research aimed to determine (1) the diversity patterns of microbial communities across ice-covered and ice-free stages; (2) the relative importance and the potential drivers of different community assembly processes across ice-covered and ice-free stages; (3) the effects of community assembly processes on different relative abundance microbes (abundant, intermediate, and rare microbes). The research was carried out in Momoge wetland, a seasonal ice-covered wetland located in Northeast China. Rhizosphere soil samples were collected throughout the ice-covered and ice-free stages. Microbial communities were characterized using the amplicon sequencing of the 16S rRNA gene (indicating bacterial communities) and ITS (indicating fungal communities). Diversity was calculated across different ice-covered stages, and the relative importance of different ecological processes was calculated using the “iCAMP” framework. The relationship between relative abundance and different ecological processes was also analyzed. We hypothesized that (1) the diversity of bacterial and fungal communities would be higher under the ice, (2) the selection processes would be influenced by the species of plants, and the dispersal limitation processes would dominate microbial communities during ice-covered stages, and (3) the rare microbial taxa would be more controlled by ecological drift than selection.

## Materials and Methods

### Study Sites and Sampling

Our study was conducted at the Momoge National Nature Reserve (45°42′25″ to 46°18′ 0″ N, 123°27′0″ to 124°4′33.7″ E), located in northeastern China. As an important hydrology node in the Nenjiang River basin (Meng, 2020), the local average annual temperature is 4.4°C, and in January, it goes down to −17.4°C. For almost half of the year, water and soil in the Momoge wetlands are entirely frozen (mid-November to mid-March) or partially frozen (late October to mid-November and mid-March to early April) (Zheng et al., 2019). However, in recent years, the Momoge wetlands were disturbed by recession flows from farmlands (Meng et al., 2019) and exhibited an abnormally high water surface during autumn and winter. This may influence the microbial diversity pattern and the community assembly processes related to the rhizosphere soil microbial community, thus obstructing bio-geographical cycling processes and plant activities during the ice-cover period (Sun et al., 2020).

We chose three sites connected by surface water but over three kilometers apart as our sample sites (30 × 30 m) (Supplementary Figure 1, see details in Supplementary Table 1). Within those three sample sites, we designed sample transects at three water depths (0, 15, and 25 cm). Along these three sample transects, we chose two emergent aquatic plant species (*Phragmites australis* (Cav.) Trin. ex Steud. and *Scirpus mucronatus* Linn.) as sample plots (0.5 × 0.5 m). In each sample plot, we sampled plant rhizosphere soil (root around 1 cm); three similar cores of rhizosphere soils were collected and mixed as one sample. Each soil sample was divided into four parts: one was used for DNA extraction and sequencing (~5 g), one for soil moisture measurements, one was freeze-dried for subsequent soil chemistry analysis, and one was stored at 4°C as a standby soil. Rhizosphere soil for DNA extraction and sequencing was saved at −20°C during transportation and stored at −80°C.

To reflect the dynamic change of the microbial communities, we set up three sample stages in the Momoge wetlands during the winter (See details in Supplementary Table 2). According to the duration of the freeze, we divided sample stages into the entirely ice-covered stage (December 2020), partially ice-covered stage (October 2020 and March 2021), and the ice-free stage (May 2021). Each stage was defined as follows: during the entirely ice-covered stage, the highest local air temperature is lower than 0°C, and water and soil are frozen throughout the whole day; during the partially ice-covered stage, the highest local air temperature is higher than 0°C while the lowest local air temperature is lower than 0°C, and the water and soil are frozen during the day; during the ice-free stage, the lowest local air temperature is higher than 0°C, and the water and soil are not frozen at any time (Supplementary Figure 2). During each sampling period, we collected 17 mixed rhizosphere soil samples, as described above (one mixed rhizosphere soil sample is missing from the expected 18, as we did not find any *Scirpus mucronatus* Linn. in Site 2 at 25 cm water depth), amounting to 68 mixed rhizosphere soil samples for all four stages.

### DNA Extraction, Sequencing, and OTU Clustering

Total genome DNA from samples was extracted using the CTAB/SDS method. The V4 region of 16S rRNA genes was amplified using a specific primer (515F and 806R) for bacteria, and the ITS2 region was amplified using a specific primer (ITS3-2024F and ITS4-2409R) for fungi. All polymerase chain reactions (PCR) contained 15 μL of Phusion® High-Fidelity PCR Master Mix (New England Biolabs), 0.2 μM of forward and reverse primers, and ~10 ng of template DNA. PCR conditions were 98°C for 1 min, followed by 30 cycles of denaturation at 98°C for 10 s, 50°C for 30 s, and 72°C for 30 s, and a final extension at 72°C for 5 min. The PCR products were mixed with the same volume of IX loading buffer (contained SYB green). The mixed PCR products underwent gel electrophoresis on 2% agarose gel for detection and were purified with a Qiagen Gel Extraction Kit (Qiagen, Germany). The library quality was assessed on the Qubit@2.0 Fluorometer (Thermo Scientific) and Agilent Bioanalyzer 2100 system. Finally, the library was sequenced on an Illumina NovaSeq PE250 platform, and 250 bp paired-end reads were generated. Paired-end reads were assigned to samples based on their unique barcode and truncated by cutting off the barcode and primer sequence. DNA extraction and sequencing were then performed at the Tianjin Sequencing Center and Clinical Lab (Beijing Novogene Technology Co., Ltd, Tianjin).

Paired-end reads were merged using FLASH (Magoč and Salzberg, 2011) to generate raw tags. Quality filtering of raw tags was performed according to Bokulich (Bokulich et al., 2013) using the QIIME (V1.9.1) (Caporaso et al., 2010). To detect chimera sequences, the 16S RNA gene tags were compared with the Silva database (version 138.1) (Quast et al., 2013; Yilmaz et al., 2014) using the UCHIME algorithm (Edgar et al., 2011); the ITS tags were compared with the UNITE database (version 8.2) (Nilsson et al., 2019) using VSEARCH (version 1.3.0) (Rognes et al., 2016). The effective tags were obtained after removing the chimera sequences detected above (Haas et al., 2011). Based on these tags, operational taxonomic units (OTUs) were clustered using the Uparse algorithm (Edgar, 2013) (Uparse version 7.0.1001) at a 97% identity threshold. Each bacterial OTU was classified against the Silva database (version 138.1) using the Mothur algorithm (Schloss, 2009). Each fungal OTU was classified against the UNITE database (version 8.2) (Abarenkov et al., 2010) using the Blast algorithm (Altschul et al., 1990). To ensure comparability, each sample was homogenized to equal sequencing depth (see details in Supplementary Table 3).

### Construction of Phylogenetic Trees

For the bacterial communities, the phylogenetic trees based on the 16S rRNA gene sequences were constructed in the FastTree software (Version 2.1.11) (Price et al., 2009) using the “maximum likelihood” method and in the Figtree software (Version 1.4.4) by setting the root using the “midpoint” method. For the fungal communities, the phylogenetic trees based on ITS sequences were constructed in the Ghost-tree software (Version 0.0.1 dev.) (Fouquier et al., 2016). This is because the ITS marker region has higher sequence variability, which not only makes ITS markers suitable for a more accurate taxonomic identification at the genus or species level but also makes the multiple sequence alignment in a long phylogenetic distance of ITS sequences highly unreliable (Fouquier et al., 2016; Tedersoo et al., 2018; Ning et al., 2020). To ensure the phylogenetic trees are robust enough for analysis based on phylogenetic distance, we applied the Ghost-tree method rather than other methods to construct the phylogenetic trees for fungi. As a construction method for hybrid-gene phylogenetic trees, the Ghost-tree constructed foundation trees according to aligned databases of the fungal 18S sequence and then grafted extension trees according to the aligned databases of the fungal ITS sequences (which have a more accurate taxonomic identification at the genus and species levels) (Fouquier et al., 2016) (Supplementary Figure 3). We chose the order level of foundation trees (equivalent to the family level of extension trees) to graft the extension trees because ITS markers are usually applied at the family or genus levels (Tedersoo et al., 2018). The extension trees at the family level contain more OTUs than the extension trees at the genus level in our Ghost-trees (Supplementary Figure 4, see details in Supplementary Table 4).

### Statistical Analysis

The alpha diversity of each sample was indicated by the observed OTUs, and the gamma diversity of a region was estimated by the Chao algorithm (Chao, 1987) using the “adiv” package (version 2.0.1) (Pavoine, 2020). Wilcoxon signed-rank tests were used to assess whether alpha diversity was significantly different between different stages and plant rhizospheres.

The community dissimilarity was calculated using the Bray-Curtis distance in the “vegan” package (version 2.5-7) (Oksanen et al., 2019). To assess the influence of distance, samples were divided into two groups: the community dissimilarity of samples within the sites and the community dissimilarity of samples among the sites. The community dissimilarity of samples within the site indicated dissimilarity over short distances (<30 m) and was referred to as “intra-site”; the community dissimilarity of samples among sites indicated dissimilarity over long distances (>3,000 m) and was referred to as “inter-sites.” The principal coordinates analysis (PCoA) based on Bray-Curtis distances was also performed on samples between two plant species. Adonis tests were undertaken to assess the difference in community dissimilarity between these two groups and plant rhizospheres. All statistical tests described were performed in the R environment (Version 4.1.1) (R Core Team, 2017).

### Analysis of Community Assembly Processes by Null Model

To determine the relative importance of different ecological processes to community assembly, we employed the “iCAMP” package. In contrast to other frameworks based on the null model, the “iCAMP” package can calculate ecological processes based on individual taxonomic groups (“bins”) rather than the entire community (Ning et al., 2020). The taxonomic groups were divided based on phylogenetic relationships between OTUs, usually containing 12~48 OTUs, which could improve the accuracy of the results of community assembly processes that are necessary for subsequent analysis at the bin level (Ning et al., 2020). This framework uses the absolute abundance OTU table and rooted phylogenetic tree to calculate the relative importance of five community assembly processes: homogeneous selection, homogeneous dispersal, dispersal limitation, heterogeneous selection (variable selection), and undominated (including ecological drift, diversification, weak selection, and/or weak dispersal; hereafter, the term ecological drift is used to represent these processes) processes. The phylogenetic signal was based on pH to determine the parameters of the phylogenetic bins: bin size (24 for bacteria; 18 for fungi) and threshold of phylogenetic distance (0.05 for bacteria; 0.025 for fungi) (Supplementary Figure 5). Then, the beta net relatedness index (βNRI) was calculated based on 1,000-times randomization of the taxa across the tips of the phylogenetic tree. The Raup–Crick metric (RC) was similarly calculated, and both used the described threshold to divide community assembly processes (Ning et al., 2020). Finally, the relative importance of ecological processes to community assembly at the subcommunity and bin levels was ascertained.

### Relationship Analysis of Microbial Rarity and Community Assembly Processes

To explore the relationship between microbial rarity and community assembly processes, bacterial and fungal communities were divided into rare, intermediate, and abundant sub-communities according to the relative abundance of phylogenetic bins. In previous studies, the sub-communities were divided according to the relative of OTUs (Wan et al., 2021). Given that there was a lower number of bins compared to OTUs (bacteria: each bin contained 24 OTUs; fungi: each bin contained 18 OTUs) and lower numbers of fungal phylogenetic bins (from 24 to 66) compared to bacterial phylogenetic bins (from 142 to 194), the sub-communities were divided based on their abundance. The bacterial communities were categorized as “rare bacterial sub-communities” when including bins with relative abundances <0.5% of the total bins; as “abundant bacterial sub-communities” when including bins with relative abundances >1% of the total bins; and as “intermediate bacterial sub-communities” for the remaining bins. To ensure that the number of bins in each sub-community was >3, the fungal communities were categorized as “rare fungal sub-communities” when including bins with relative abundances <1% of the total bins; as “abundant fungal sub-communities” when including bins with relative abundances >5% of the total bins; and as “intermediate fungal sub-communities” for the remaining bins. We calculated the ratio of the ecological processes of the three microbial sub-communities to total ecological processes. The relative importance of each of the five ecological processes in these sub-communities, i.e., homogeneous selection, homogeneous dispersal, ecological drift, dispersal limitation, and heterogeneous selection (variable selection) processes, was determined using ‘bin_contribution_to_each_process’ (*BP*_τ*k*_) data. To calculate community assembly processes at the bin level, the relationship between the relative importance of different ecological processes in governing each bin (*P*_τ*k*_) and the relative abundance of each bin were analyzed by Spearman's rank correlation. The relative abundance of all bins (explanatory variables), the value of which was equal to zero, was deleted.

## Results

### Alpha, Gamma, and Beta Diversity Analysis of Microbial Community

**Figure 1:**
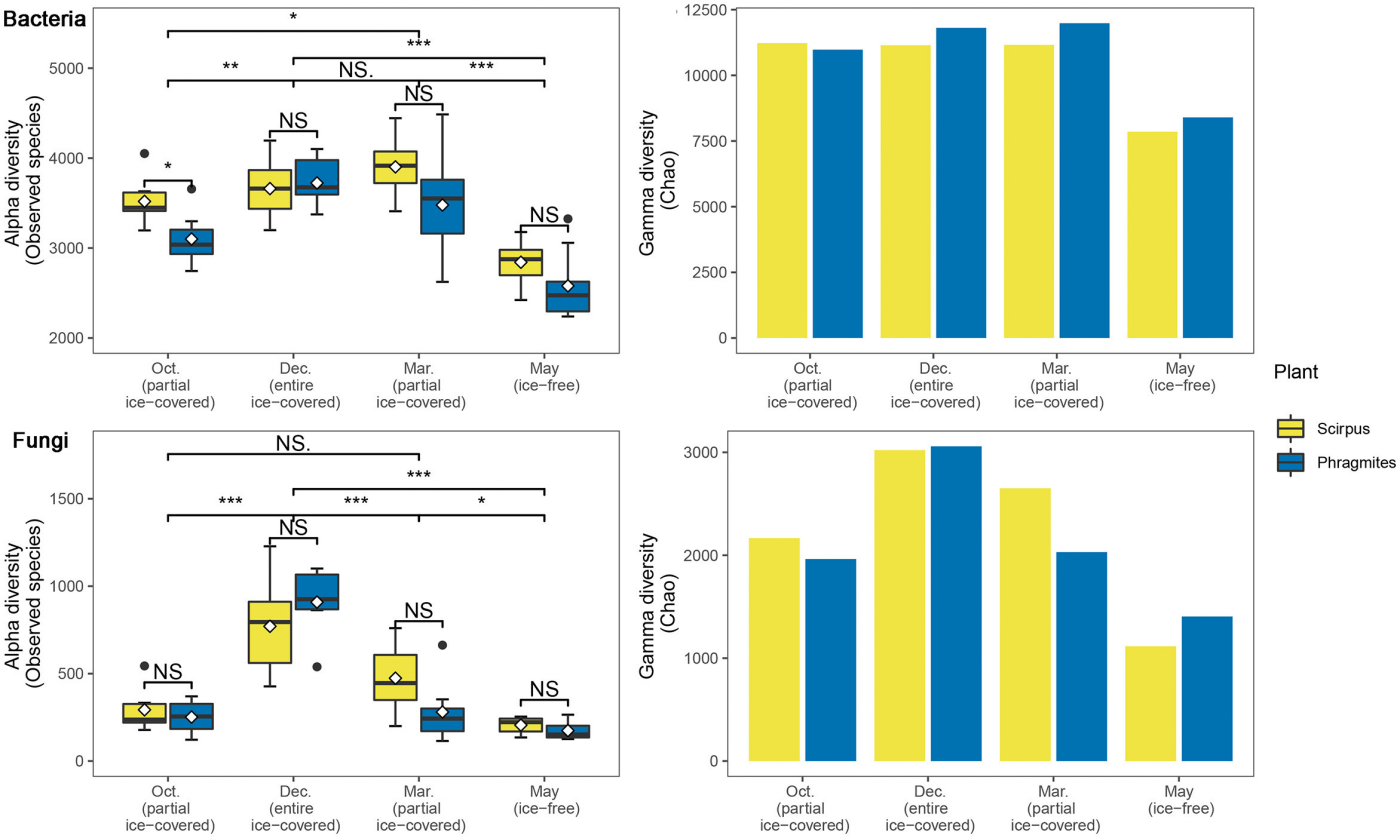
Alpha and gamma diversities of bacterial and fungal communities during different ice-covered stages. Significance of the Wilcoxon signed-rank test: NS., not-significant; *p < 0.05; **p < 0.01; ***p < 0.001.

The alpha diversity of the bacterial communities during the ice-covered stage was greater than that during the ice-free stage (22.4–36.7%, *p* < 0.05) (Figure 1). Similarly, the alpha diversity of the fungal communities during the ice-covered stage was higher than that during the ice-free stage (45.9–341.5%, *p* < 0.05), and was more significant than the diversity pattern of the bacteria communities. The gamma diversity of the bacterial community during the ice-covered stage was also greater than that during the ice-free stage (31.1–35.9%). Similarly, the gamma diversity of the fungal community during the ice-covered stage was more than that during the ice-free stage (82.4–100.4%). In the other plant rhizosphere habitats, most of the alpha and gamma diversity of microbial communities did not show a significant difference during the same stage and showed a similar change between different stages. Only in the first partially ice-covered stage (October 2020) did the alpha diversity of bacterial communities differ significantly between the two rhizosphere habitats.

**Figure 2:**
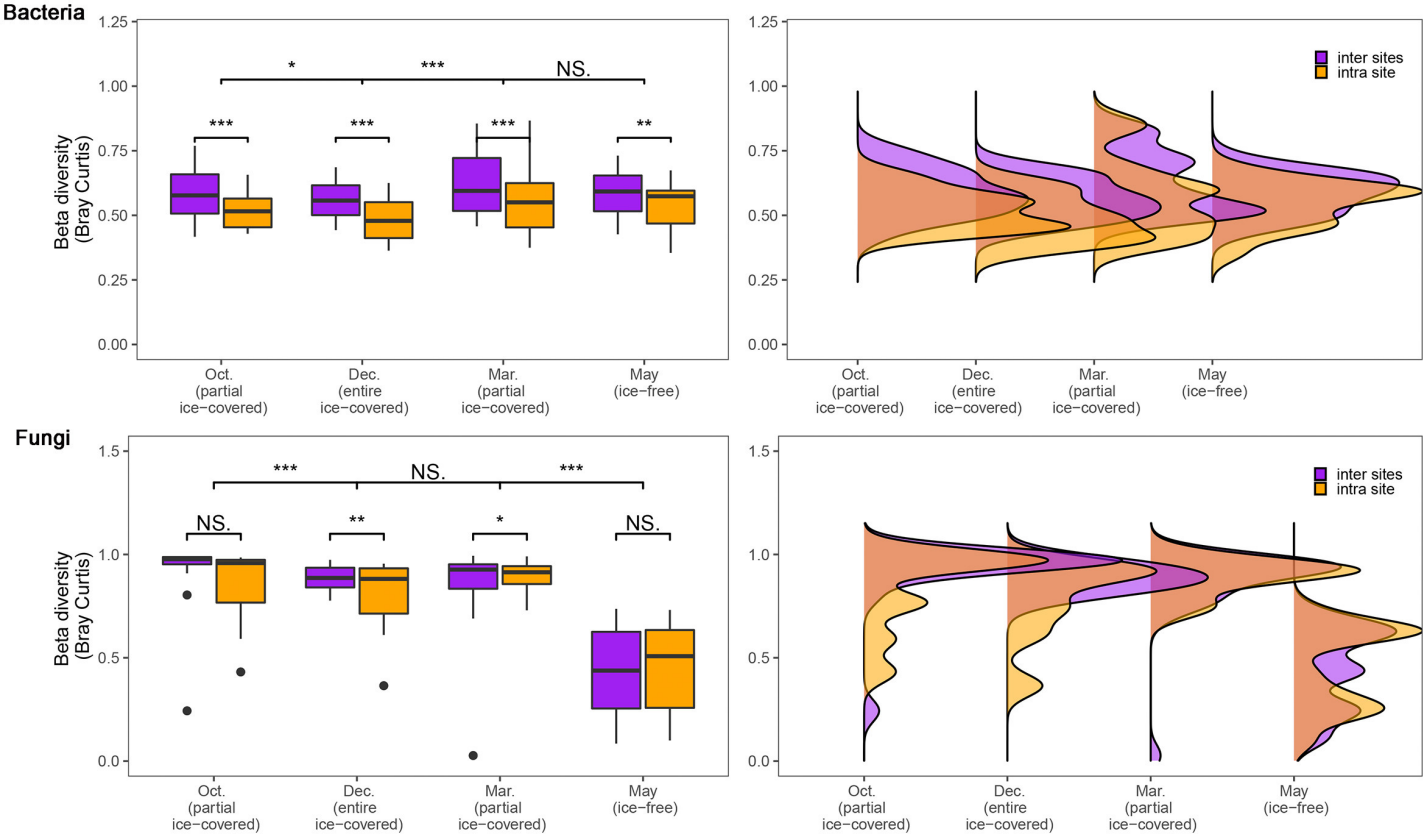
Dissimilarity of bacterial and fungal communities during different ice-covered stages. Community dissimilarity was calculated using the Bray-Curtis distance. To assess the influence of geographical distances, the samples were divided into “inter sites” and “intra site” groups. The “inter site” group includes samples among different sites (>3,000 m); the “intra site” group includes samples within the same site (range of 30 × 30 m). Significance of the Adonis tests: NS., not-significant; *p < 0.05; **p < 0.01; ***p < 0.001.

To assess the influence of geographical distance on the microbial communities, the community dissimilarity (calculated by Bray-Curtis distance) between intra-sites and inter-sites was calculated and tested using the Wilcoxon signed-rank test (Figure 2). The dissimilarity of bacterial communities was significantly lower in intra-sites compared to inter-sites (*p* < 0.05) during all stages, with no exceptions. However, the dissimilarity of the fungal communities showed different patterns during the ice-free stage and other stages. During entirely and partially ice-covered stages, the dissimilarity of the fungal communities was significantly lower in intra-sites than in inter-sites (*p* < 0.05). During the ice-free stage, the dissimilarity of the fungal communities showed no significant difference either in intra-sites or in inter-sites. The dissimilarity of the fungal communities during the ice-free stage was significantly lower than at any other stage (*p* < 0.05).

### Microbial Community Assembly Processes During Different Ice-Covered Stages

**Figure 3:**
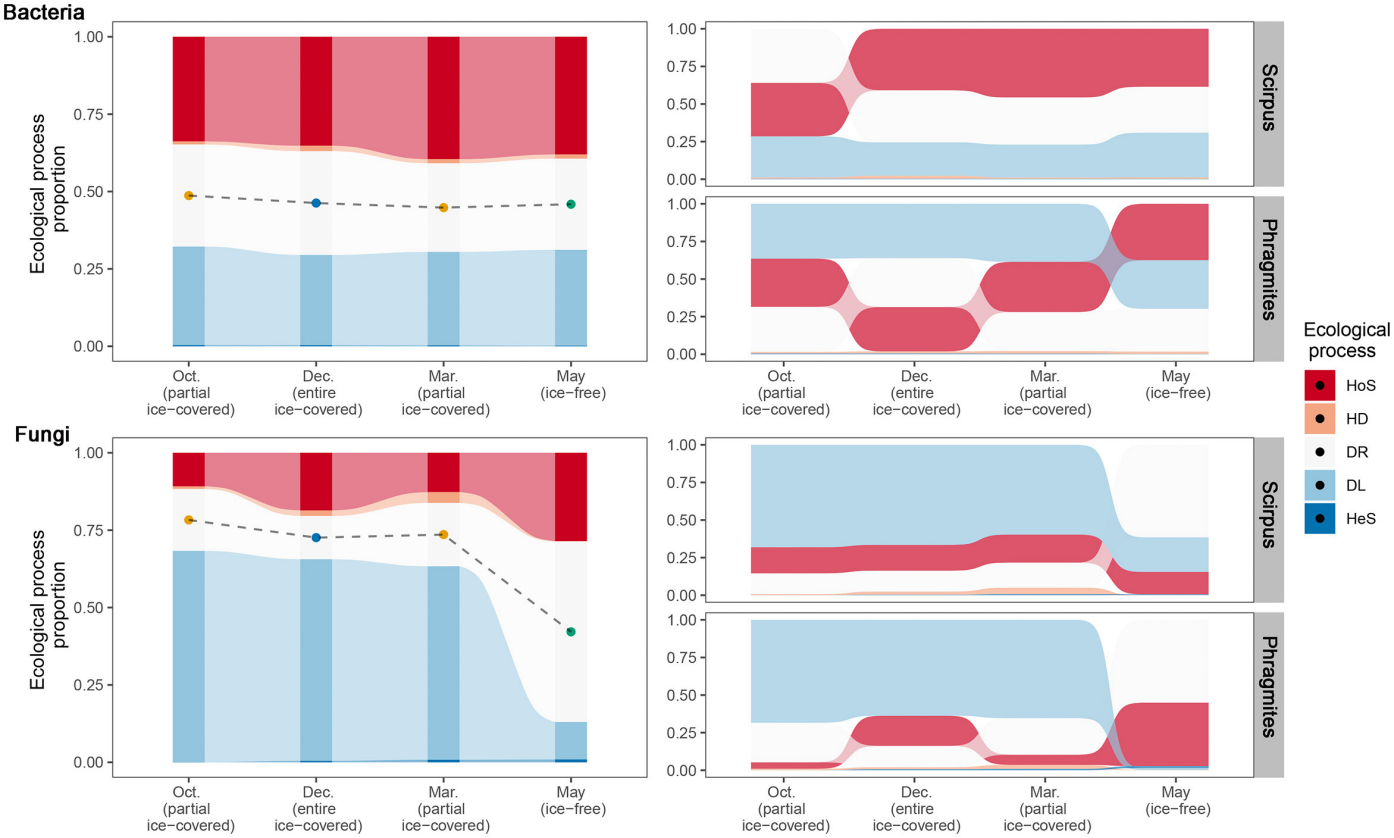
Relative importance of different ecological processes to bacterial and fungal communities across different ice-covered stages and plant species. Different fill colors indicate different community assembly processes: HoS, homogeneous selection; HD, homogeneous dispersal; DR, ecological drift (i.e., undominated processes, including drift, diversification, weak selection, and/or weak dispersal); DL, dispersal limitation; HeS, heterogeneous selection. In the two left sub-figures, points depict the midpoint of the ecological drift process and reflect the deviation compared to the null model. In the two sub-figures on the right, processes were ranked according to the relative importance (proportion) from top to bottom to reflect the turnover of dominant processes across different ice-covered stages.

To reveal the community assembly mechanism during different ice-covered stages, ecological processes and their relative importance in the microbial communities were calculated (Figure 3). The results calculated by the “iCAMP” package showed that the main ecological processes of bacterial community assembly were homogeneous selection, dispersal limitation, and ecological drift. The main ecological processes of fungal community assembly were dispersal limitation, ecological drift, and homogeneous selection.

The turnover of different ecological processes during various ice-covered stages for the bacterial communities was minor. In contrast, for the fungal communities, there was a distinct shift between different stages. From entirely and partially ice-covered stages to the ice-free stage, the main ecological processes of the fungal community assembly changed from dispersal limitation (from 62.5–68.3 to 12.1 %) to homogeneous selection (from 10.8–18.6 to 28.5%) and ecological drift (from 14.0–20.5 to 58.3%). This means that during entirely and partially ice-covered stages, the fungal communities tended to be heterogeneous between different samples (dispersal limitation) and that during the ice-free stage, they tended to be homogeneous (homogeneous selection). Therefore, the community assembly processes of fungi were more influenced by the ice-covered environment or seasons than the community assembly processes of bacteria.

The turnover of different ecological processes was also different in the different plant rhizospheres. In the rhizosphere habitat of *Phragmites* (*Phragmites australis* (Cav.) Trin. ex Steud.), the proportion of homogeneous selection processes increased in both bacterial and fungal communities during the ice-free stage. In contrast, in the rhizosphere habitat of *Scirpus* (*Scirpus mucronatus Linn*.), the proportion of different ecological processes in the bacterial communities during various ice-covered stages was stable. However, the ecological drift process increased to the highest magnitude in the fungal communities during the ice-free stage.

### Relationship Between Ecological Processes and Abundance of Microbial Subcommunities (Rare, Intermediate or Abundant) in Various Ice-Covered Stages

**Figure 4:**
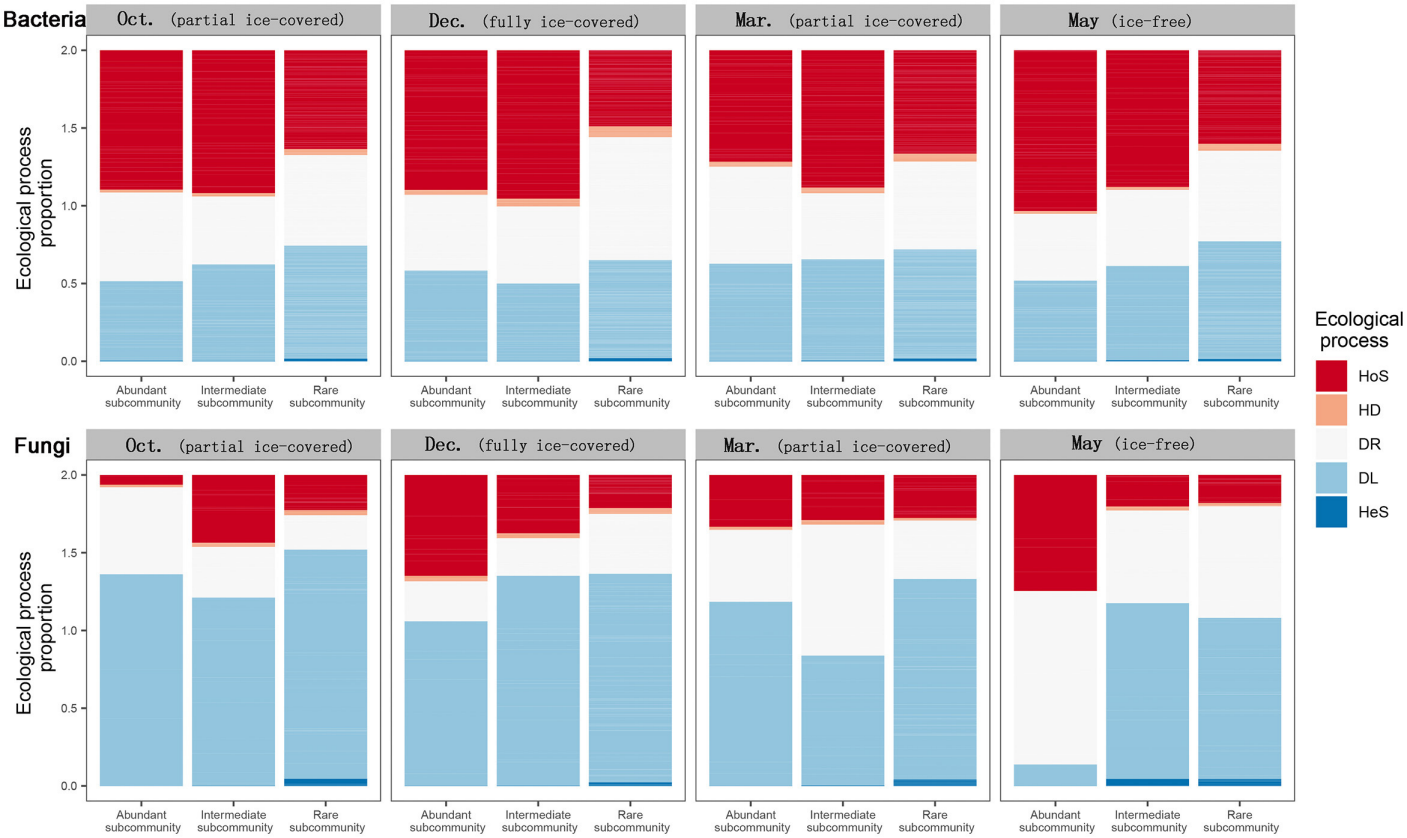
Relative importance of different ecological processes to abundant, intermediate, and rare bacterial and fungal subcommunity assemblies during different ice-covered stages. Different fill colors indicate different community assembly processes: HoS, Homogeneous Selection; HD, Homogeneous Dispersal; DR, ecological drift (i.e., undominated processes, including drift, diversification, weak selection, and/or weak dispersal); DL, Dispersal Limitation; HeS, Heterogeneous Selection.

To explore the relationship between microbe rarity and the ecological processes of community assembly, the impact of the ecological processes on different microbial subcommunities (including rare, intermediate, and abundant microbial subcommunities) was calculated (Figure 4). During all stages in the bacterial communities, rare microbial subcommunities were more controlled by dispersal limitation than homogenous selection; however, the opposite was true for abundant subcommunities. In contrast, no such pattern was apparent for the fungal communities between rarity and ecological processes. During entirely and partially ice-covered stages, dispersal limitation was the dominant process for rare, intermediate, and abundant fungal microbial subcommunities. On the other hand, during the ice-free stage, abundant fungal subcommunities tended to be influenced by homogenous selection and ecological drift, while fungal intermediate and rare subcommunities tended to be influenced by dispersal limitation and ecological drift.

### Relationship Between Ecological Processes and Abundance of Microbial Bins in Various Ice-Covered Stages

**Figure 5:**
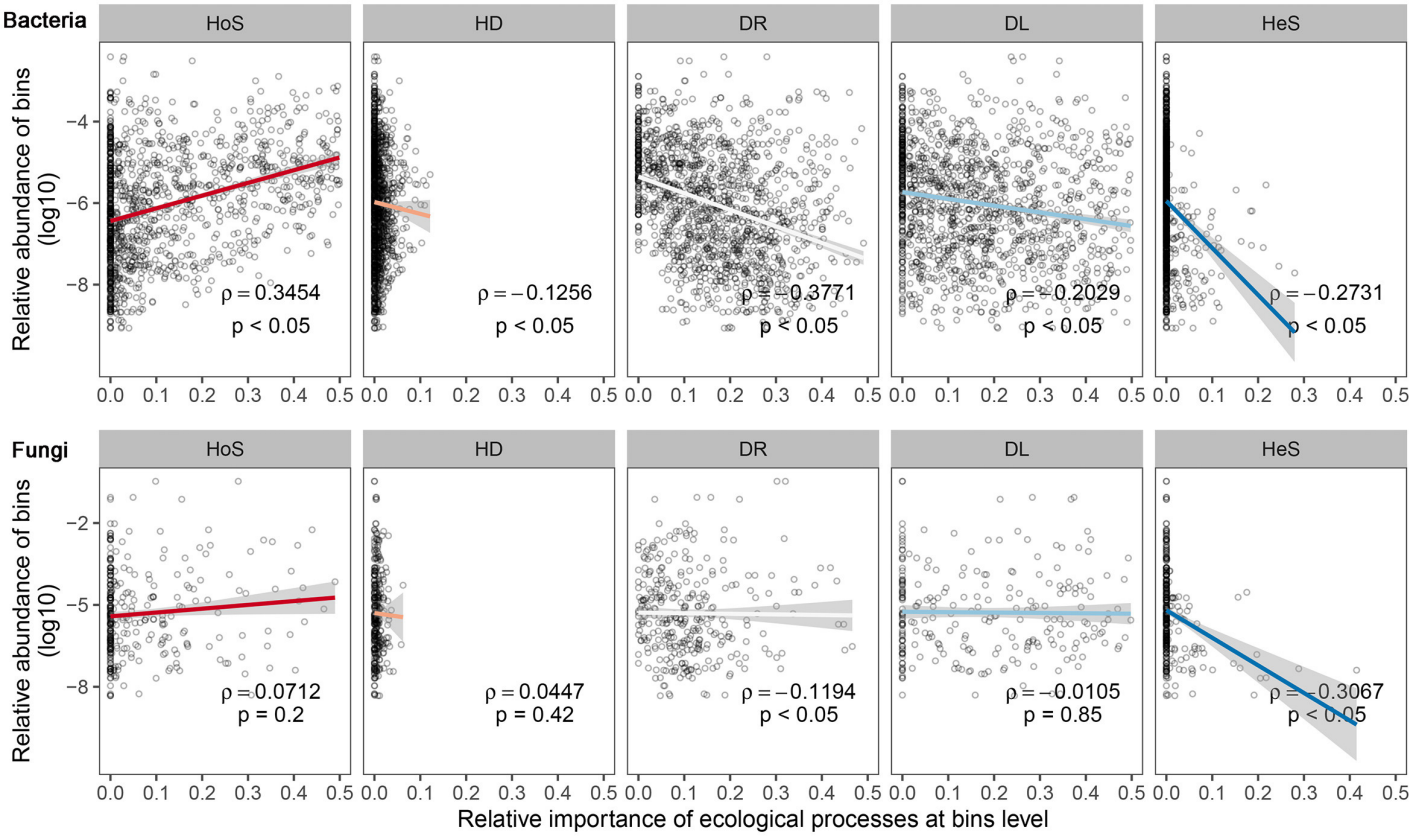
Relationship of relative abundance of bacterial and fungal community taxa bins and their ecological processes of assembly at the bin level. Different sub-captions indicate different community assembly processes: HoS, homogeneous selection; HD, homogeneous dispersal; DR, ecological drift (i.e., undominated processes, including drift, diversification, weak selection, and/or weak dispersal); DL, dispersal limitation; HeS, heterogeneous selection. The horizontal axis depicts assembly processes' relative importance per bins' relative abundance; and the vertical axis is the bins' relative abundance, which has been transformed by logarithmic conversion. For each sub-figure, we add a tendency line (ggplot2 package, “lm” method); 95% confidence interval of the tendency line is depicted by shading. Significance of Spearman's rank correlation: p, coefficient of Spearman's rank correlation: ρ.

To verify the pattern of relative abundance and the ecological processes of microbial bins, Spearman's rank correlation was employed to test the relationship between the relative abundance of microbial bins and the relative importance of different ecological processes in governing each bin (Figure 5).

Interestingly, this result shows that ecological processes affect microbial bins of varying relative abundance in a different way for both bacterial and fungal communities. The relative importance of most processes for each bin was significantly correlated (*p* < 0.05) to the abundance of each bin. This means that if a microorganism belongs to an abundant microbial subcommunity (or a rare microbial subcommunity), it tends to be influenced by certain community assembly processes rather than others. For bacterial communities, the homogeneous selection tended to exert control more on a relatively high abundance microbe rather than on a low abundance microbe (i.e., rare microbe). Moreover, the homogeneous selection, ecological drift, dispersal limitation, and heterogeneous selection processes tended to have more of an influence on low abundance than on high abundance bacteria. Compared to bacteria, the relationship between the fungal community assembly processes and the relative abundance had a similar direction but lower strength. Moreover, there was a significant correlation between the relative importance of ecological drift and heterogeneous selection processes for each OTU.

## Discussion

### Higher Microbial Community Diversity Under the Ice

The diversity pattern of microbial communities under the ice, especially in fungal communities, is still unclear. In this study, the bacterial communities under the ice were characterized by a slightly higher alpha and gamma diversity (Figure 1). Furthermore, the alpha and gamma diversity for fungal communities under the ice exhibited patterns similar to the bacterial communities, and the pattern was even more significant than the bacterial diversity pattern (Figure 1). Previous studies in north wetlands suggested that soil contains more nitrogen and phosphorus during the ice-covered stage, which may be caused by the lower nutrient requirements and higher litter inputs of plants during the non-growth season (Gao, 2021). This could provide numerous niches and increase the diversity of microorganisms. Moreover, compared to bacteria, fungi are considered to have a higher resistance to low temperatures. A controlled experiment using the phospholipid fatty acid analysis reveals that fungi grow faster at sub-zero temperatures just as bacteria grow faster at above-zero temperatures (Haei et al., 2011). Other studies also support that fungi have higher diversity in frozen soil without any cover (Chen et al., 2021; Sang et al., 2021). Therefore, the higher resistance of fungi to low-temperature environments could improve the diversity of fungi under the ice in this study.

### Different Temporal Dynamics of Bacterial and Fungal Community Assembly Processes

Community assembly processes shape the diversity and biogeography of microorganisms. Quantifying the relative importance of different processes could help us identify the main drivers of microbial communities across ice-covered and ice-free wetlands.

In this study, we used the “iCAMP” model to infer the relative importance of bacterial and fungal communities across ice-covered and ice-free stages. In this study, bacterial communities were mainly influenced by homogeneous selection during both ice-covered and ice-free stages, while fungal communities were mainly influenced only during the ice-free stage rather than during the ice-covered stage (Figure 3). Homogeneous selection includes host filtering. Host filtering of plants in the rhizosphere habitat may be one driver for the turnover of fungal communities between stages. May is the growing season of *Phragmites* and *Scirpus* when the photosynthesis of these two plants is at its peak. This may lead to a stronger filter of roots on fungi than would be possible during the non-growing season (He et al., 2020). Therefore, homogeneous selection controlled more fungi in the ice-free stage compared to the ice-covered stage. Previous studies suggest that plant roots could filter the microbes in specific taxa and decrease the community dissimilarity (Edwards et al., 2015; Trivedi et al., 2020). The dissimilarity of the fungal communities sharply decreased from the ice-covered stage to the ice-free stage, which also provides indirect evidence that the increase in plant host filtering shapes the fungal communities in the ice-free stage (Figure 2; Supplementary Figure 8). Previous studies showed that *Phragmites* have stronger host activities in the ice-free stage (Fang et al., 2021) and milder host effects in the ice-covered stage (Gao, 2021) compared to other general wetland plants. These are consistent with our result that fungal and bacterial communities in *Phragmites* were more controlled by homogeneous selection in ice-free stages (Figure 3). Our study also showed that compared to *Phragmites, Scirpus* had a similar host effect during the ice-covered stage (Supplementary Figure 7) but milder host effects on fungi and bacteria in the growth stage (Figure 3; Supplementary Figure 7).

Our “iCAMP” result also showed that the dispersal limitation process of the fungal communities, but not of the bacterial communities, had a sharp decrease from the ice-covered to the ice-free stage (Figure 3). Water, in liquid form, is considered to be an ideal medium for the dispersal of microorganisms (Lindström and Langenheder, 2012). The main propagules of fungi are spores or sporocarps, which could disperse in water for a long distance because water, in its liquid form, could prevent them from spore desiccation and ultraviolet light damage (Golan and Pringle, 2017). In this study, the dispersal medium across ice-covered and ice-free stages changed from ice to water, and this may have facilitated the dispersal of fungi *via* spores and therefore decreased the dispersal limitation. The disappeared difference between intra-site and inter-site dissimilarity of fungal communities in the ice-free stage also implies that water, in its liquid form, may have facilitated the dispersal of fungi (Figure 2). Since the propagules of most bacteria are themselves, compared to other microorganisms dispersal through spores, these bacteria are more easily limited by harsh abiotic conditions in long-distance dispersal (Yang and van Elsas, 2018). Moreover, bacteria could also disperse *via* fungal hyphae (Yang and van Elsas, 2018), which could have kept the dispersal of bacteria immune to the change of the abiotic medium in our research (Figure 3). This study provides indirect evidence that the change of the dispersal medium drives the spatial variation pattern of the fungal communities between ice-covered (water in solid form) and ice-free (water in liquid form) stages. In future, the underlying mechanisms need to be further verified by more direct evidence, including observations in natural ecosystems and controlled experiments.

### Different Effects of Community Assembly Processes on Various Abundance Levels of Microorganisms (Both at the Subcommunity Level and Bin Level)

Previous research has generally studied the relationship between microbial abundance and community assembly processes at the subcommunity level, which could only provide qualitative information. Moreover, this relationship has not been researched in ice-covered wetlands. In this study, we used the “iCAMP” model to investigate this relationship in ice-covered wetlands, both at subcommunity and bin levels.

Our result at subcommunities levels suggested that, during the ice-free stage, abundant fungal subcommunities were more controlled by homogenous selection and the ecological drift process, while intermediate and rare fungal subcommunities were more controlled by dispersal limitation and the ecological drift process (Figure 4). Homogeneous ecological processes (including homogeneous dispersal and homogeneous selection) tended to dampen the community dissimilarity (Jia et al., 2018). The sharp decrease in the relative importance of dispersal limitation to abundant fungal subcommunities may have caused the dampened dissimilarity such that the intra-site and inter-site fungal communities were more similar. This result implies that the abundant fungal subcommunities, rather than the rare fungal subcommunities, dampened the dissimilarity of fungal communities during the ice-free stage in this study.

Our result at bin levels demonstrates that the dispersal limitation process showed a slightly higher tendency to control rare microbes, while homogeneous selection highly influenced abundant microbes. Compared to research in other ecological zones, research in bays (Mo et al., 2018) and wastewater (Lee et al., 2021) found similar patterns. Research in terrestrial ecosystems found that homogeneous selection greatly influenced rare microbes (Jiao and Lu, 2020; Zheng et al., 2021). The different niche breadths of rare microbes may be the reason for the different conclusions in different studies. Arguably, the narrow niche breadths of microbial taxa allow those taxa to be influenced by homogeneous selection (Jiao and Lu, 2020). In our study, rare microbes tended to have intermediate niche breadths rather than narrow ones, and abundant microbes tended to have both narrow and wide niche breadths (Supplementary Figure 6). Consequently, abundant microbes, rather than rare microbes, tended to be greatly influenced by homogeneous selection. In some studies where rare microbes had narrow niches, they were greatly controlled by homogeneous selection. Another explanation is the dormancy of rare microbes. Under the ice-covered surface, the environmental temperature was low such that some of the microorganisms were in dormancy or exhibiting low growth rates. Dormancy dampens the strength of homogeneous selection and leads to rare microbial taxa but will not cause extinction. It will also cause the homogeneous selection to influence abundant microbes rather than rare microbes. Those tendencies exist in bacterial and fungal communities equally, albeit they are stronger in bacterial communities. The possible explanation is that, during the ice-covered stage, the growth rate of abundant and rare microbes differed to a larger extent. That is, some microbes were active, had faster growth rates, and were more likely to be abundant, but were also more likely to be influenced by homogeneous environmental selection. However, some microbes exhibited very low activity (e.g., microorganisms in a dormant state), had lower growth rates, and were more likely to be rare but less influenced by homogeneous environmental selection. In our study, community assembly processes such as homogeneous selection, ecological drift, or dispersal limitation had a specific relationship with the relative abundance of microbial OTUs. Although diversity and assembly processes of the microbial community were influenced by external environmental factors, such as periods of ice-coverage, the presence of plants, and the dispersal medium, the pattern seemed to be steady and not influenced by these factors (Supplementary Figure 9). It might be valuable to verify whether this pattern is widespread in microbial communities and to further investigate the underlying mechanisms of this pattern.

## Conclusion

Our study suggests that fungal communities have higher alpha diversity under the ice. Homogeneous selection, dispersal limitation, and ecological drift dominated the assembly of bacterial communities during the ice-covered stages and ice-free stages. Dispersal limitation dominated the assembly of fungal communities during the ice-covered stages. Host filtering mainly affected the assembly of fungal communities during the ice-free stage in the Phragmites rhizosphere. In our research, we found that abundant microbes were controlled more by homogeneous selection, while rare microbes were controlled more by ecological drift, dispersal limitation, and heterogeneous selection, especially for bacteria. In future, more directed observation and controlled experiments are required to comprehensively assess these conclusions.

## Data Availability Statement

The data presented in the study are deposited in the National Center for Biotechnology Information (NCBI) repository, accession number PRJNA813903.

## Author Contributions

JM provided concept, designed methodology, performed the field sampling, conducted the data analyses and wrote the manuscript. KM performed the field sampling and reviewed this manuscript. JL supervised this research and reviewed this manuscript. KM, JL, and NC assisted to methodology designing. All authors contributed to the article and approved the submitted version.

## Funding

This research was supported by the National Key Research and Development Program of China (No. 2016YFC0500402).

## Conflict of Interest

The authors declare that the research was conducted in the absence of any commercial or financial relationships that could be construed as a potential conflict of interest.

## Publisher's Note

All claims expressed in this article are solely those of the authors and do not necessarily represent those of their affiliated organizations, or those of the publisher, the editors and the reviewers. Any product that may be evaluated in this article, or claim that may be made by its manufacturer, is not guaranteed or endorsed by the publisher.
