# Supplementary material for: Rhizosphere Soil Microbial Community Under Ice in a High-Latitude Wetland: Different Community Assembly Processes Shape Patterns of Rare and Abundant Microbes
Source: Front Microbiol. 2022 May 23;13:783371. doi: 10.3389/fmicb.2022.783371 (PMC9169045; doi:10.3389/fmicb.2022.783371)
Supplement: Supplementary file 1 [file Data_Sheet_1.docx]

Supplementary Material

Jiaming Ma*, Kang Ma, Jingling Liu*, Nannan Chen

## Supplementary Figures

**
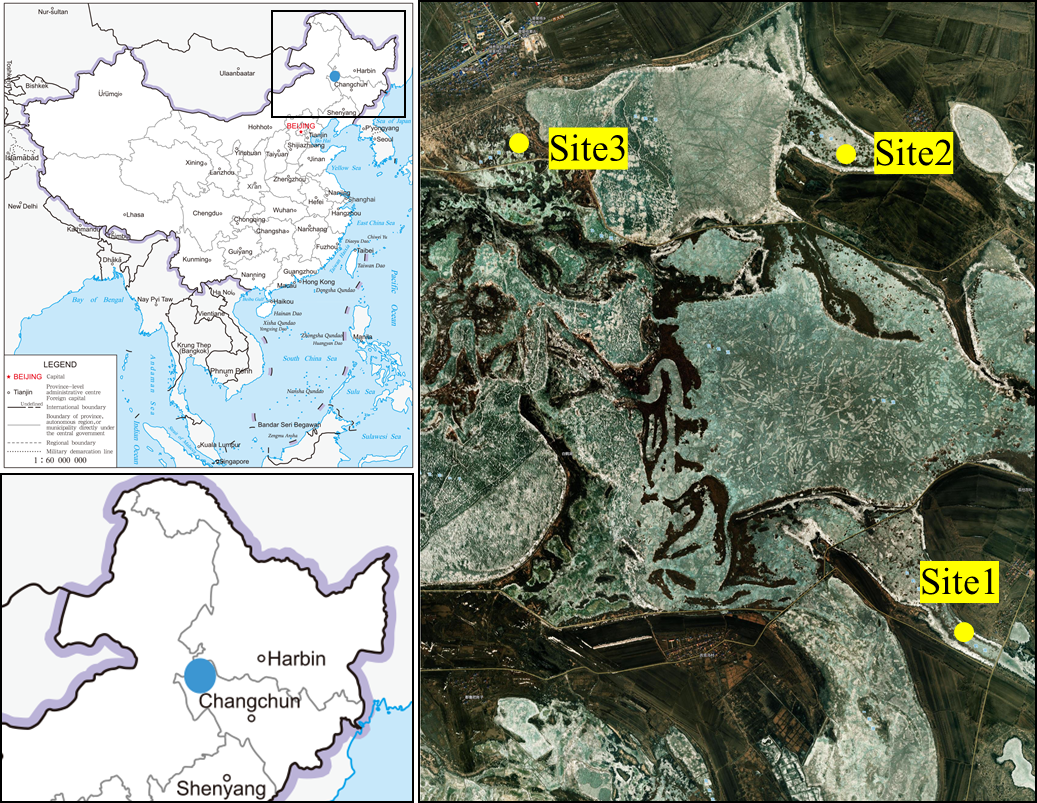
FIGURE S1 |** Momoge wetland is located in northeast China. Sampling sites in Momoge wetland were apart for over 3,000 meters.


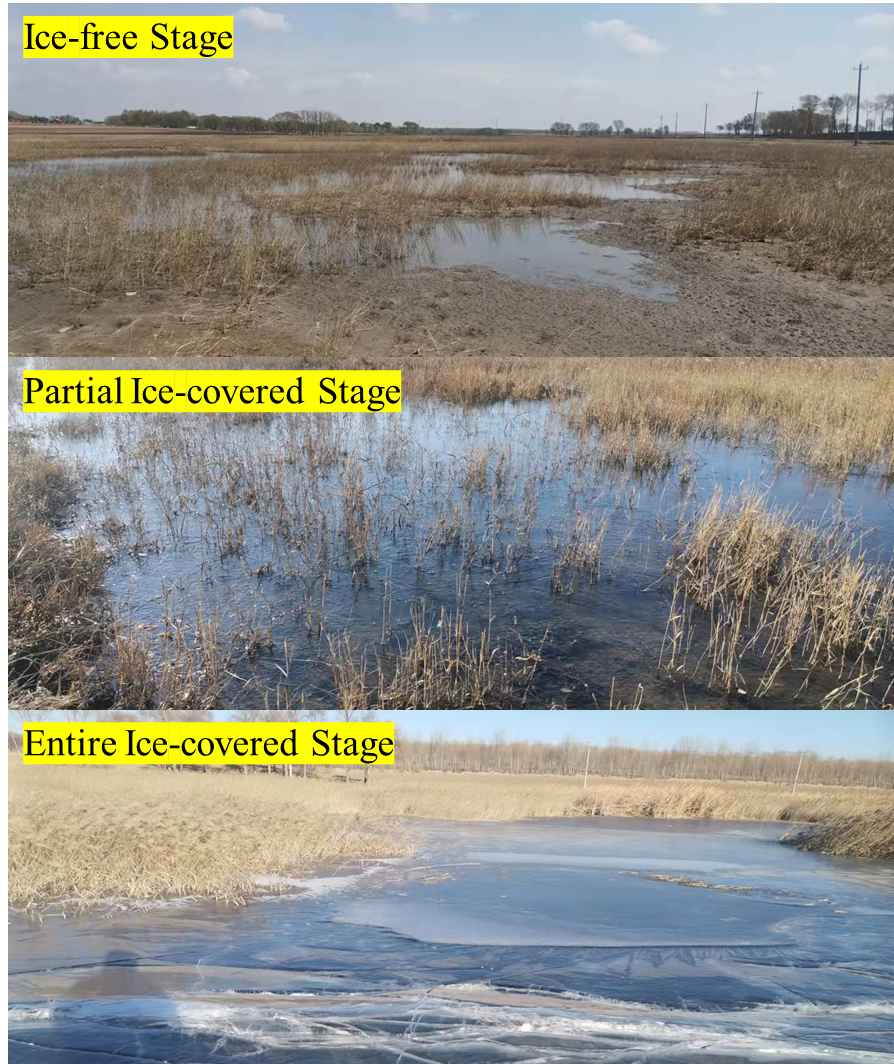
**FIGURE S2 |** Environment Change during ice-free, partial ice-covered, and ice-covered stages in Momoge wetland. During the ice-free stage, the lowest air temperature is higher than 0℃ and water is liquid; during the partial ice-covered stage, the lowest air temperature is lower than 0℃, the highest air temperature is higher than 0℃, and topwater is frozen while bottom water is liquid; during the entire ice-covered stage, the highest air temperature is lower than 0℃, and whole water is freeze.


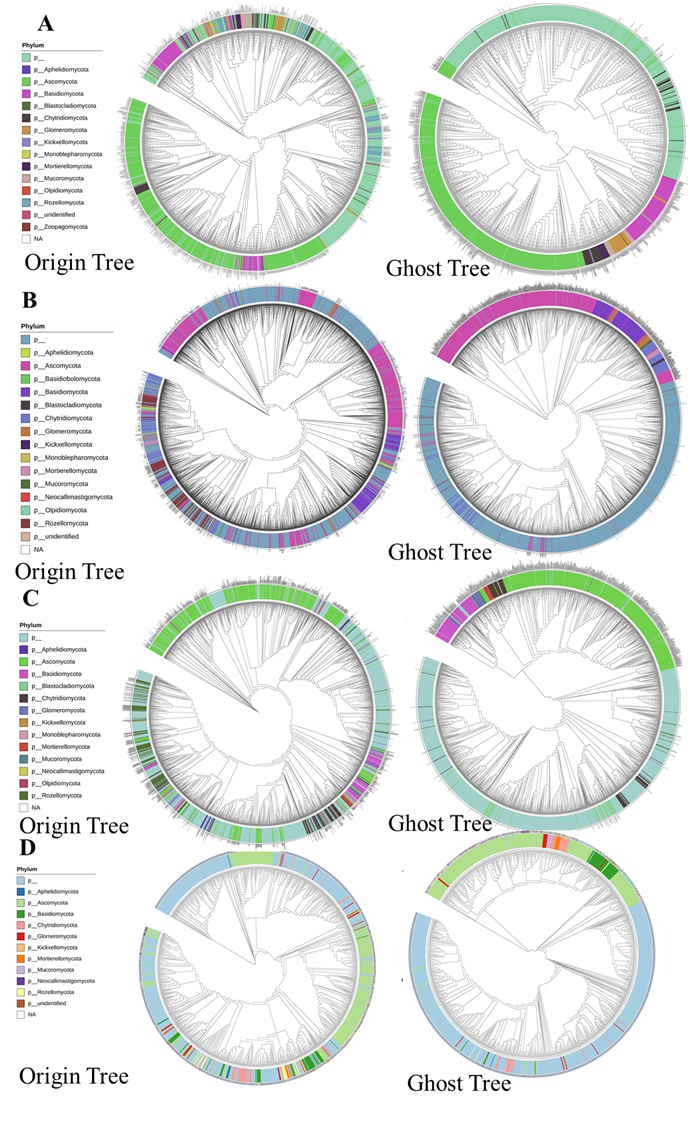


FIGURE S3 | Comparison of phylogenetic trees based on ITS sequencing results. (A) Samples were in October 2020. (B) Samples collected in December 2020. (C) Samples were collected in March 2021. (D) Samples were collected in May 2021. In each sub-figure, the ‘Origin Tree’ is a phylogenetic tree using Fasttree to construct and ignored the unreliable ITS markers. The ‘Ghost tree’ are phylogenetic trees using Ghost-tree to construct (graft at order level of the foundation trees) and applied the hybrid-gene phylogenetic trees construction method to dampen the unreliable of ITS markers.


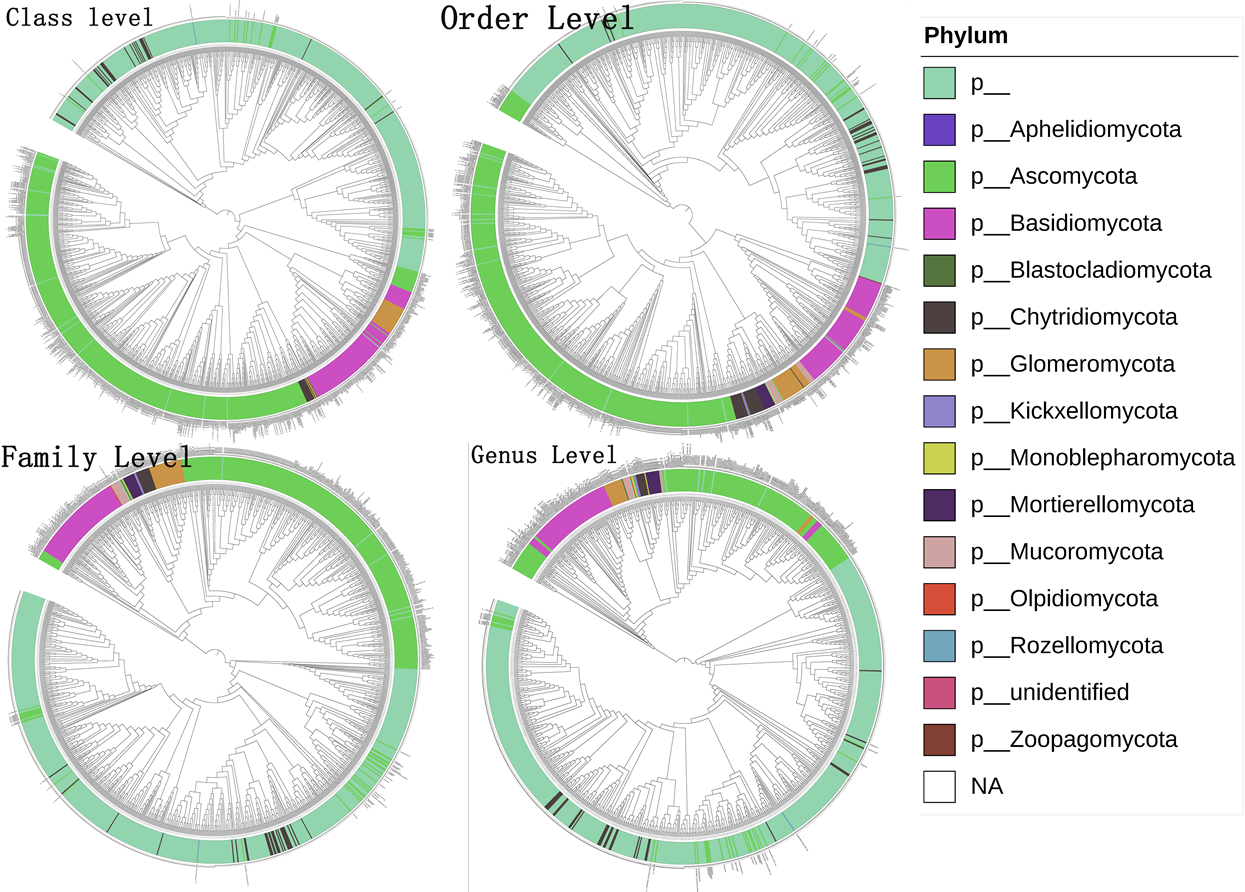


FIGURE S4 | Comparison of phylogenetic trees constructed at different graft levels using the ghost-tree method. These phylogenetic trees were constructed based on the ITS sequencing results sampled in October 2020.


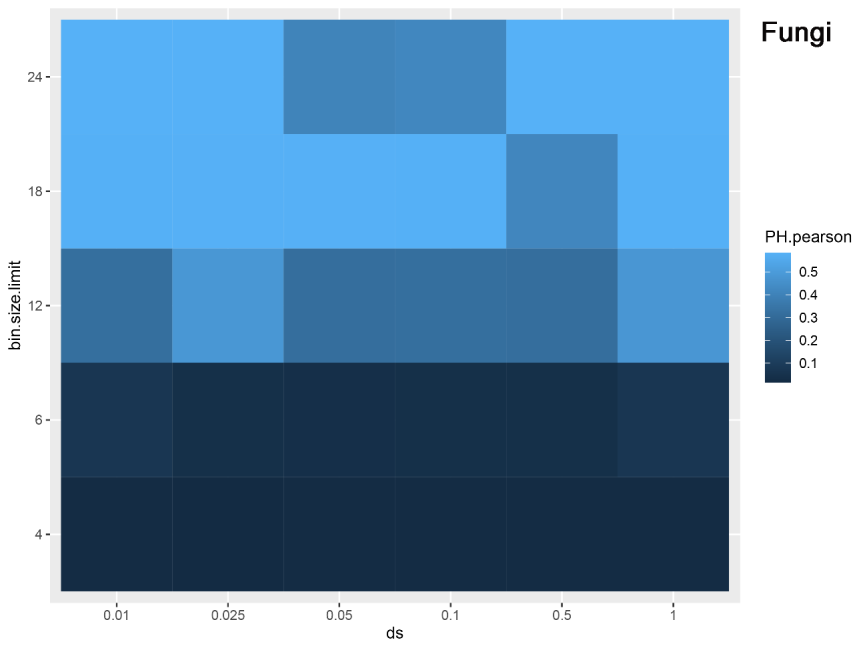

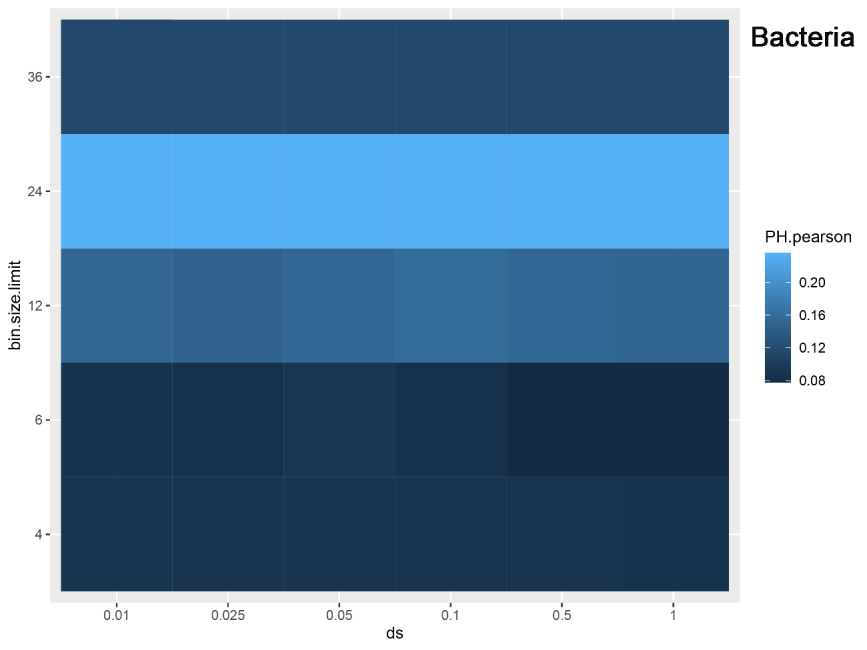
**FIGURE S5 |** Phylogenetic signal based on PH. “ds” means phylogenetic distance, while “bin.size.limit” means the amounts of OTUs in one phylogenetic bin at most.

**
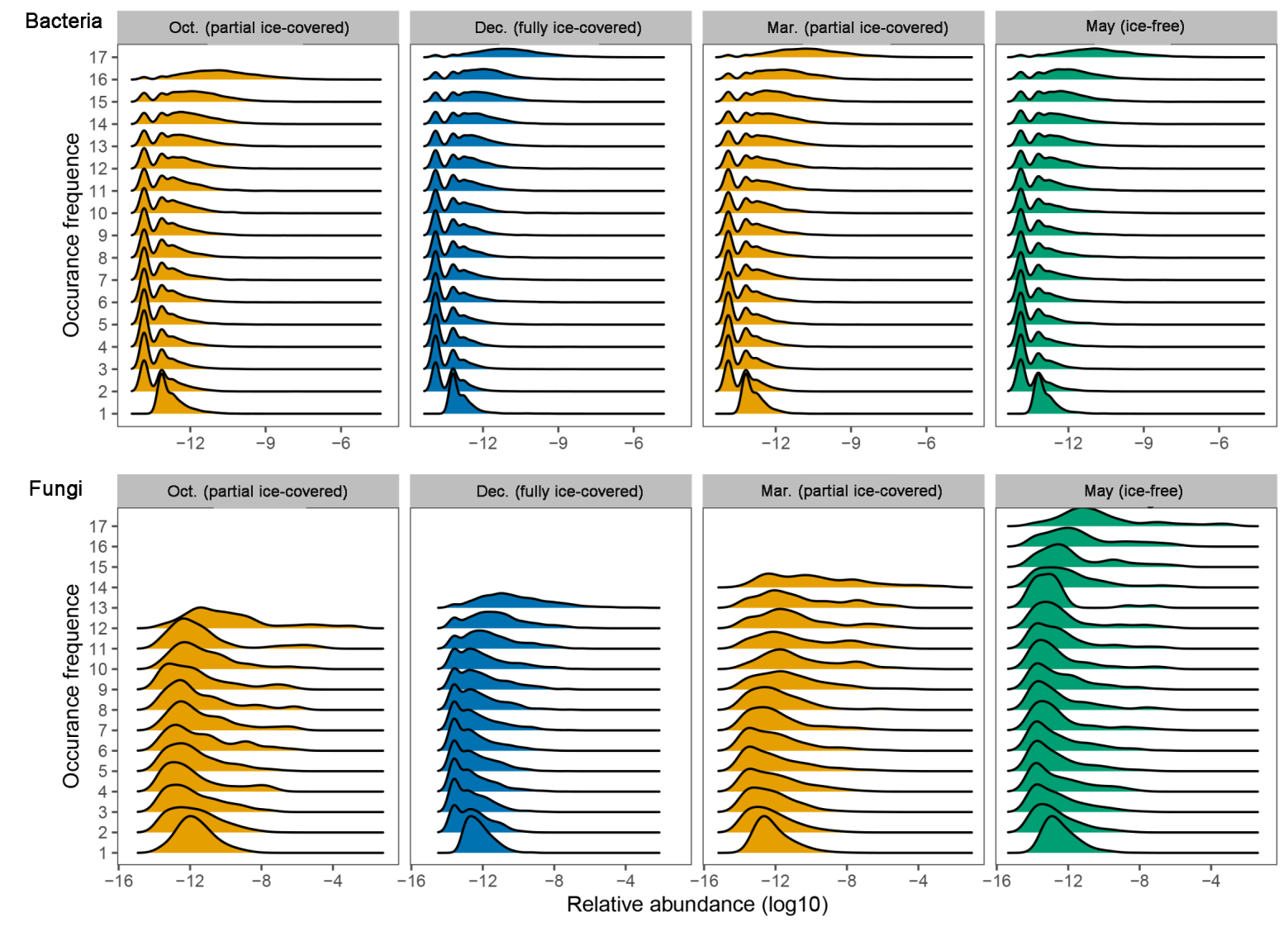
FIGURE S6 |** Occurrence-abundance relationship of the bacterial community (16S rRNA gene) and fungal community (ITS rRNA gene). The horizontal axis “log10 (abundance)” means OTUs relative abundance, which has been transformed by logarithmic conversion. The vertical axis is the occurrence frequency of OTUs in all samples. (In several sub-figures, the max occurrence frequency is lower than 17, that’s because some samples were not performed by high-throughput sequencing due to technical reasons.)


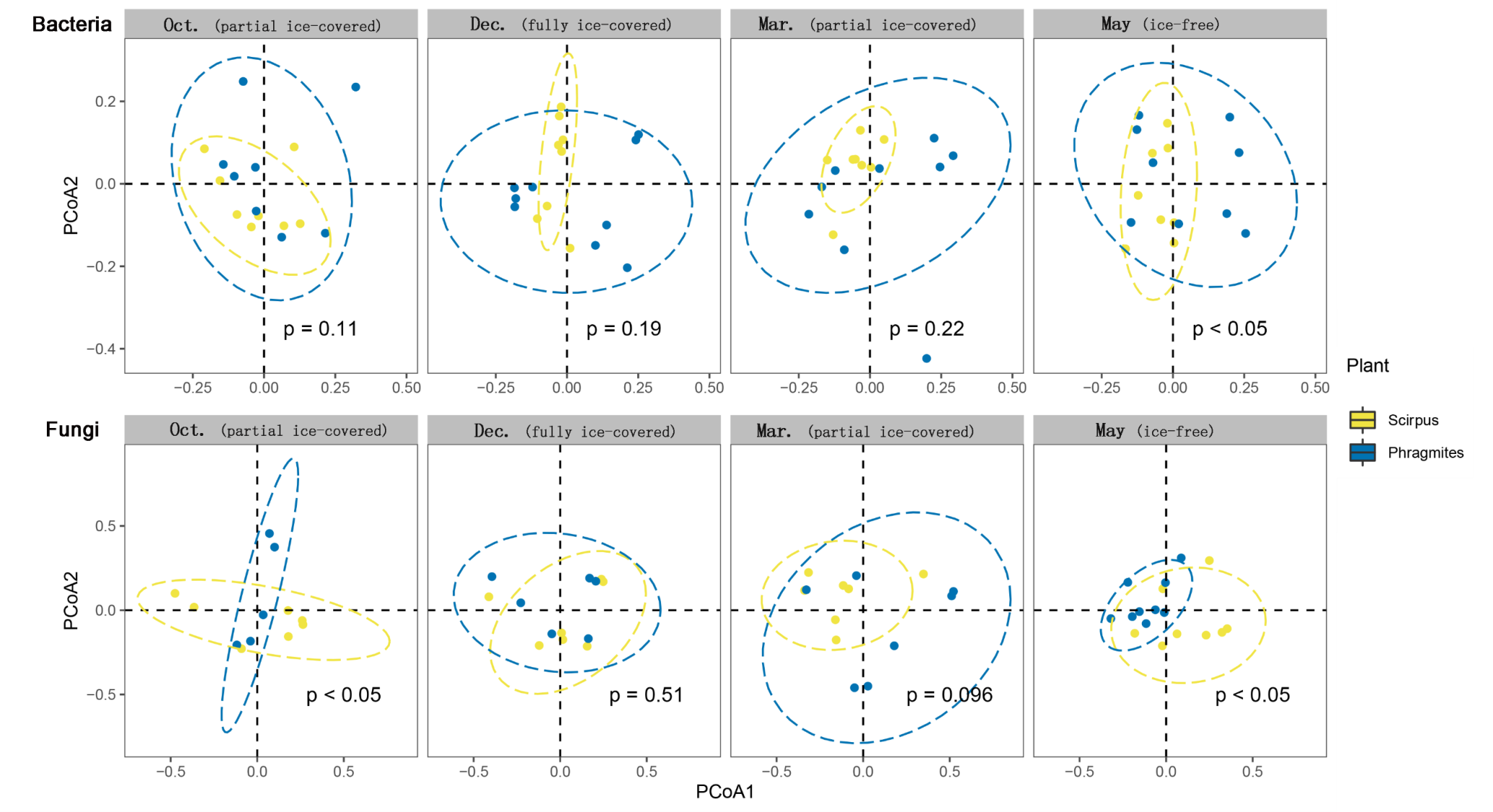


**FIGURE S7 |** The principal coordinates analysis (PCoA) based on Bray ­Curtis distances between two plants. “Scirpus” indicates the rhizosphere of *Scirpus mucronatus Linn.*, and the “Phragmites” indicate the rhizosphere of *Phragmites australis* (Cav.) Trin. ex Steud. test


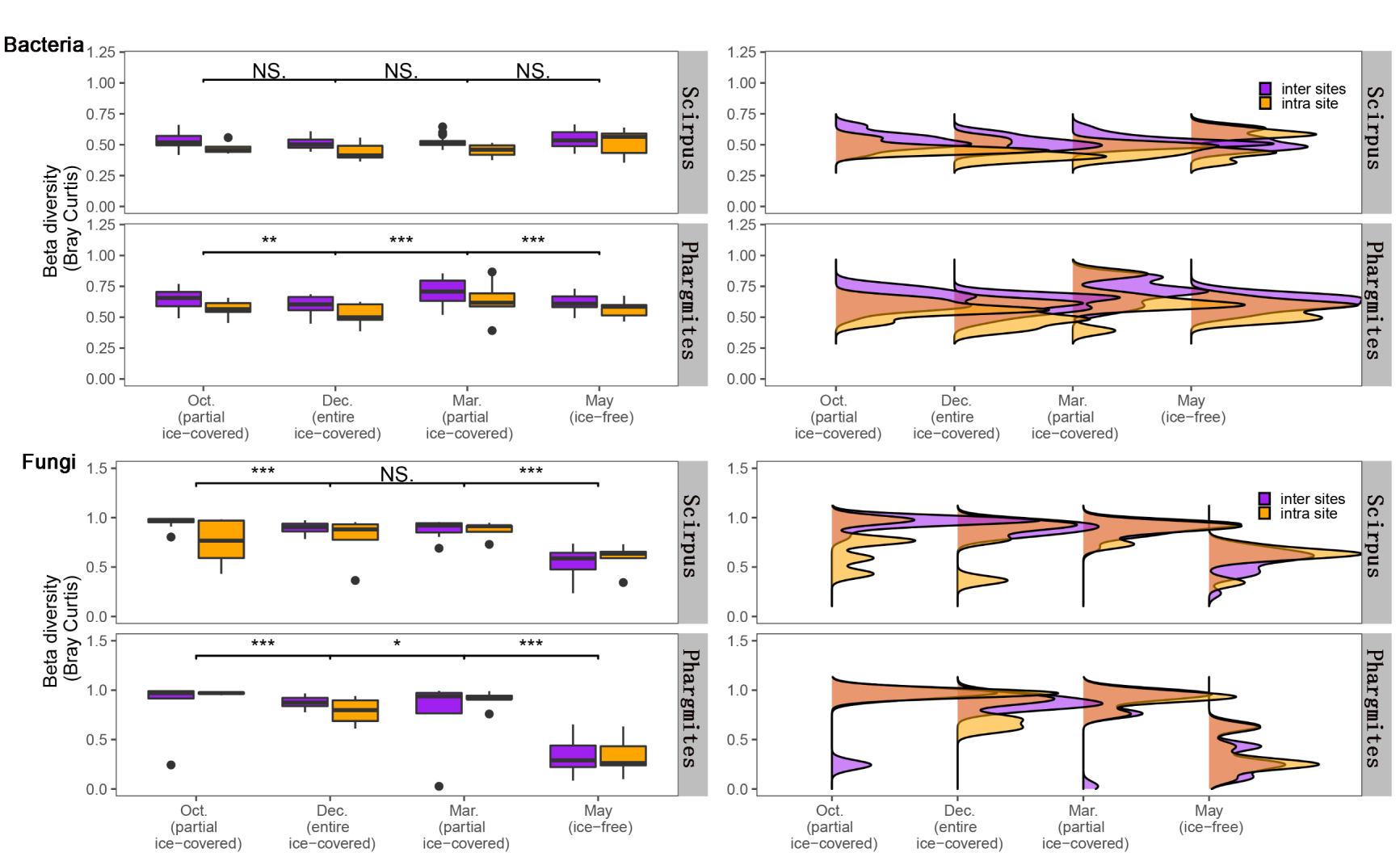


**FIGURE S8 |** Dissimilarity of bacterial and fungal communities based on the Bray-Curtis distance during different ice-covered stages and different plant rhizosphere. To assess the influence of geographical distances, the beta diversity is divided into “inter sites” and “intra site” groups. The “inter sites” group includes samples among different sites (> 3,000 m); the “intra site” includes samples within the same site (range of 30 $\boldsymbol{\times}$ 30 m). The “Scirpus” indicates the rhizosphere of *Scirpus mucronatus Linn.*, and the “Phragmites” indicate the rhizosphere of *Phragmites australis* (Cav.) *Trin. ex Steud.*


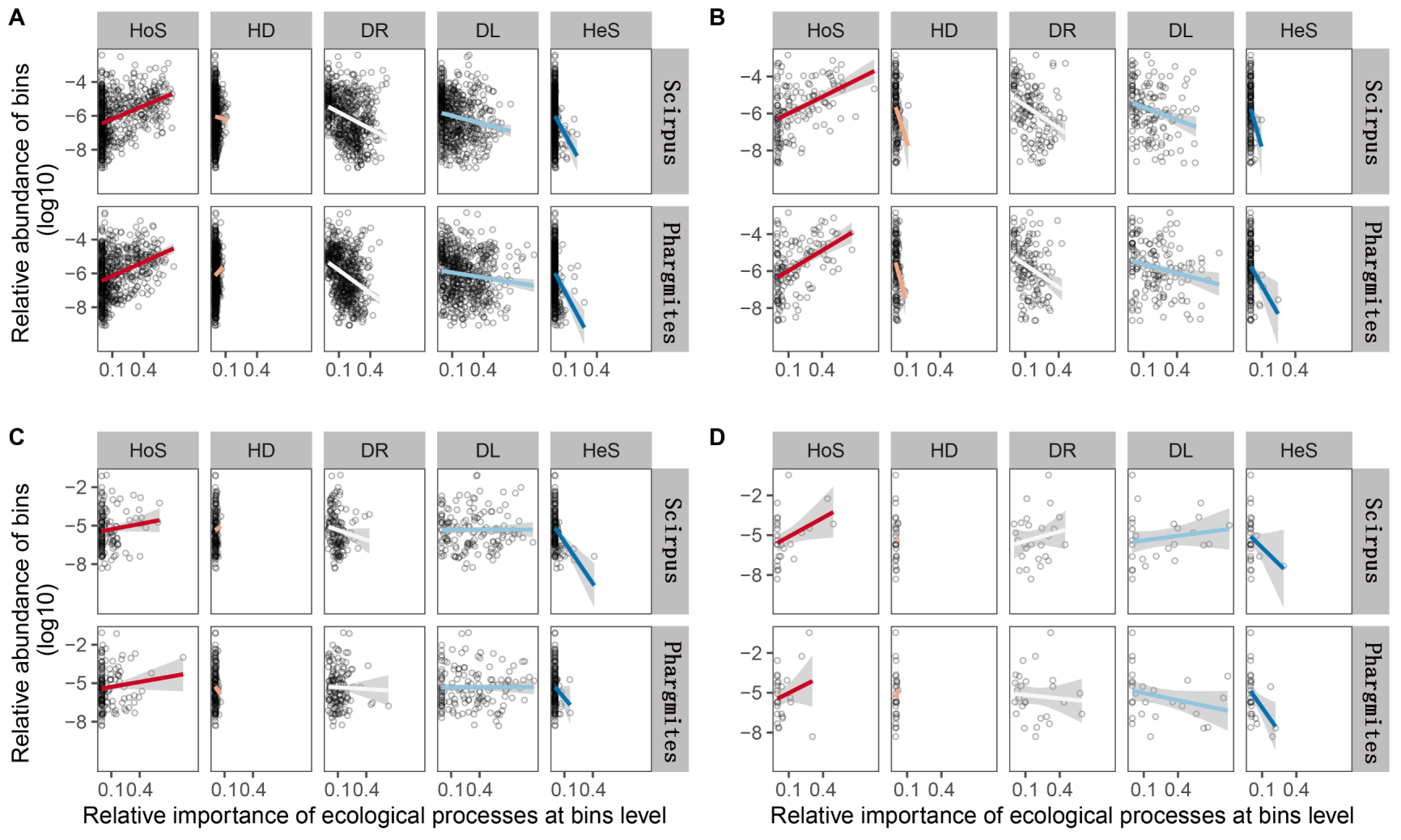


**FIGURE S9 |** Relationship of relative abundance of bacterial and fungal community taxa bins and their assembly processes. **(A)** Bacterial samples collected in October 2020, December 2020 and March 2021. **(B)** Bacterial samples collected in May 2021. **(C)** Fungal samples collected in October 2020, December 2020 and March 2021. **(D)** Fungal samples collected in May 2021. In each sub-figure, Different sub-captions indicate different community assembly processes: HoS, Homogeneous Selection; HD, Homogeneous Dispersal; DR, Ecological Drift; DL, Dispersal Limitation; HeS, Heterogeneous Selection. The horizontal axis depicts assembly processes’ relative importance per bin relative abundance; and the vertical axis is the bin’s relative abundance, which has been transformed by logarithmic conversion. For each sub-figure, we add a tendency line (ggplot2 package, “lm” method); the 95 % confidence interval of the tendency line is depicted by shading.

## Supplementary Tables

TABLE S1 | Geographical Coordinates of Sampling Sites

| **Site** | **longitude** | **latitude** |
| --- | --- | --- |
| Site1 | 123° 41′ 24″ | 45° 53 ′ 29″ |
| Site2 | 123° 40′ 27″ | 45° 56′ 11″ |
| Site3 | 123° 37 ′37″ | 45° 56′ 14″ |

TABLE S2 | Sampling Time

| **Stage** | **Sampling Time** |
| --- | --- |
| Stage1 | October 27th, 2020 ~ October 30th, 2020 |
| Stage2 | December 7th, 2020 ~ December 9th, 2020 |
| Stage3 | March 22nd, 2021 ~ March 24th, 2021 |
| Stage4 | May 1st, 2021~ May 3rd, 2021 |

TABLE S3 | Sequencing Depth after Homogenized

| **Kingdom** | **Stage** | **Sequencing Depth** |
| --- | --- | --- |
| Bacteria (16S rRNA gene) | Oct. | 55792 |
| Bacteria (16S rRNA gene) | Dec. | 58676 |
| Bacteria (16S rRNA gene) | Mar. | 58322 |
| Bacteria (16S rRNA gene) | May | 59091 |
| Fungi (ITS rRNA gene) | Oct. | 59616 |
| Fungi (ITS rRNA gene) | Dec. | 55013 |
| Fungi (ITS rRNA gene) | Mar. | 49163 |
| Fungi (ITS rRNA gene) | May | 59849 |

Table S4 | The number of OTUs contained in ghost-tree at different foundation tree’s graft level

| Stage | Class | Order | Family | Genus |
| --- | --- | --- | --- | --- |
| October 2020 | 1343 | 1332 | 1146 | 965 |
| December 2020 | 2401 | 2485 | 2193 | 2024 |
| March 2021 | 1842 | 1822 | 1646 | 1527 |
| May 2021 | 975 | 952 | 876 | 833 |
